# Global Epidemiology and Mechanisms of Resistance of *Acinetobacter baumannii-calcoaceticus* Complex

**DOI:** 10.1093/cid/ciad109

**Published:** 2023-05-01

**Authors:** Mariana Castanheira, Rodrigo E Mendes, Ana C Gales

**Affiliations:** JMI Laboratories, North Liberty, Iowa, USA; JMI Laboratories, North Liberty, Iowa, USA; Division of Infectious Diseases, Department of Internal Medicine, Escola Paulista de Medicina (EPM), Universidade Federal de São Paulo (UNIFESP), São Paulo, Brazil

**Keywords:** epidemiology, resistance mechanisms, international clones, *A. baumannii-calcoaceticus* complex

## Abstract

*Acinetobacter baumannii-calcoaceticus* complex is the most commonly identified species in the genus *Acinetobacter* and it accounts for a large percentage of nosocomial infections, including bacteremia, pneumonia, and infections of the skin and urinary tract. A few key clones of *A. baumannii-calcoaceticus* are currently responsible for the dissemination of these organisms worldwide. Unfortunately, multidrug resistance is a common trait among these clones due to their unrivalled adaptive nature. *A. baumannii-calcoaceticus* isolates can accumulate resistance traits by a plethora of mechanisms, including horizontal gene transfer, natural transformation, acquisition of mutations, and mobilization of genetic elements that modulate expression of intrinsic and acquired genes.

## Teaser

*Acinetobacter baumannii-calcoaceticus* complex (ABC) became an important group of organisms causing human infections worldwide. A small number of clones are responsible for the increased number of infections and resistance to numerous antimicrobial agents. We describe the current epidemiology and resistance mechanisms in ABC.

The genus *Acinetobacter* was initially proposed by Brisou and Prevot in 1954 [1], but it was not accepted until after the Baumann, Doudoroff, and Stanier study of 1968 [2]. The Subcommittee on the Taxonomy of *Moraxella* and Allied Bacteria recognized the genus in 1971 [3] and, by 1974, it was included in the *Bergey's Manual of Systematic Bacteriology* [4].

The *Acinetobacter* genus includes gram-negative, strictly aerobic, indole-negative, nonfastidious, nonmotile, catalase-positive, and oxidase-negative bacteria that are citrate positive with a DNA guanine-cytosine (G + C) content of 39–47% [5]. At the time of its initial description, the genus included a single species, *Acinetobacter calcoaceticus* [4]. Since then, the taxonomy of the genus *Acinetobacter* has undergone significant modifications as additional species are included and its nomenclature updated. To date, 74 *Acinetobacter* species have been accepted [6]. Unfortunately, *Acinetobacter* isolates at the species level are not easily identified due to phenotypic and genotypic similarities. Molecular tests are often necessary for correct identification [7]. For this reason, clinically significant *Acinetobacter* species, such as *A. baumannii*, *A. nosocomialis*, *A. pittii*, *A. seifertii*, and *A. lacticae* (also called *A. dijkshoorniae*) [6], as well as the environmental species *A. calcoaceticus*, are collectively designated members of the so-called *Acinetobacter baumannii-calcoaceticus* complex (ABC) [5, 8].

Among the ABC species, *A. baumannii* is the most common cause of human infections [7–9]. Although community-acquired infections caused by *A. baumannii* are reported in hot and humid countries, these infections are uncommon [10]. In contrast, infections are commonly reported in the nosocomial setting, especially intensive care units (ICUs) worldwide. These infections include ventilator-associated pneumonia and catheter-related bloodstream and surgical site infections [11]. Risk factors for *A. baumannii* infection include admission to the ICU, advanced age, immunosuppression, intravascular devices, invasive procedures, malignancies, mechanical ventilation, prior colonization, prolonged hospital stay, recent surgery, and severity of illness [9, 12]. In addition, the use of carbapenems, third-generation cephalosporins, and broad-spectrum penicillins was also associated with an increased risk of nosocomial-acquired pneumonia by multidrug-resistant *A. baumannii* [12, 13].

## GLOBAL EPIDEMIOLOGY

*Acinetobacter baumannii* has globally emerged as a highly important pathogen in healthcare settings due to its ability to resist desiccation, develop tolerance to biocides, and survive with minimal nutrient requirements. As such, *A. baumannii* has a remarkable ability to acquire or upregulate various resistance determinants [11, 14]. While carbapenems are one of the β-lactams with the broadest spectrum and highest in vitro activity against gram-negative bacilli and used to be an excellent option for the treatment of *Acinetobacter* spp. infections, increasing resistance rates have jeopardized their clinical utility.

However, carbapenem susceptibility rates vary according to the geographic region. Among *Acinetobacter* spp. collected between 2013 and 2016 by the SENTRY Antimicrobial Surveillance Program, the lowest susceptibility rates for meropenem were observed for Latin America (13.7%), followed by the Asia-Pacific region (21.0%), Europe (22.2%), and the United States (54.9%) [15]. Among *Acinetobacter* spp. collected between 2016 and 2018 by Seifert and colleagues [16], the lowest susceptibility rates for meropenem were observed for Africa and the Middle East (17.2%), followed by Latin America (19.6%), Asia–South Pacific (31.4%), Europe (33.8%), and North America (63.6%). In the latest World Health Organization (WHO) and European Centre for Disease Prevention and Control (ECDC) report, the percentages of carbapenem-resistant *Acinetobacter* spp. varied widely within Europe in 2020: of 38 countries and areas reporting data, 3 countries reported occurrence rates of less than 1%, whereas 35 reported rates of 50% or higher [17]. The countries with the lowest rates were Ireland, the Netherlands, and Norway, whereas carbapenem resistance rates were at or above 50% in 21 countries, mostly in Southern and Eastern Europe.

Colistin resistance was uncommon during 1990s; the first resistance case was reported from the Czech Republic in 1999 [18]. According to the SENTRY Antimicrobial Surveillance Program, a significant reduction in colistin susceptibility rates was noticed for all geographic regions in 2013–2016 when compared with the 2005–2008 data. The highest decrease in susceptibility to colistin in ABC was observed in Europe (to 89.6% from 99.2%), followed by the Asia-Pacific region (to 93.7% from 99.1%) and North America (to 93.6% from 98.4%) [15].

Based on initial molecular typing of *A. baumannii* isolates, 3 lineages of *A. baumannii* disseminated in Europe. These lineages were classified as European clones I, II, and III [19, 20]. Once further studies showed that these lineages had already spread globally, European clones I, II, and III were renamed international clonal (IC) lineages I, II, and III [20, 21]. To date, 9 ICs of *A. baumannii*, ICs 1–9, have been recognized [22]. Later, 2 multilocus sequence type (MLST) schemes, Oxford and Pasteur, were defined for the characterization of *A. baumannii* isolates, and different schemes generate different sequence types (STs) and clonal complexes [23, 24]. The correlation among *A. baumannii* international clones as assessed by Oxford and Pasteur MLST schemes are *A. baumannii* IC1 (CC109^OXF^/CC1^Past^), IC2 (CC92^OXF^/CC2^Past^), IC3 (CC929^OXF^/CC124^Past^), IC4 (CC103^OXF^/CC15^Past^), IC5 (CC227^OXF^/CC79^Past^), IC6 (CC944^OXF^/CC78^Past^), IC7 (CC110^OXF^/CC25^Past^), IC8 (CC447^OXF^/CC10^Past^), and IC9 (CC1078^OXF^/CC464^Past^) [21, 22, 24, 25]. IC1 and IC2 commonly harbor the acquired carbapenemase oxacillin-hydrolysing (OXA)-23, and these ICs are the most successfully disseminated clones worldwide [26, 27]. However, regional variations occur. IC5 and IC7 are more predominant in Central and South America [20], whereas IC9 is more predominant in Africa and the Middle East [22]. Unfortunately, it is difficult to draw an accurate picture of the spread of *A. baumannii* clones due to the lack of publicly available genome sequence data from Africa, the Middle East, South America, and Russia [26]. A strong correlation between *bla*_OXA-51_–like sequences and *A. baumannii* ICs 1–8 was reported, suggesting that *bla*_OXA-51_ sequencing could be applied as a simple test to identify *A. baumannii* ICs [28].

In the United States, an estimated 8500 cases and 700 deaths were related to carbapenem-resistant *Acinetobacter* spp. infections in 2017. These numbers decreased in 2018 (6300 cases, 500 deaths), remained stable in 2019 (6000 cases, 500 deaths), but increased in 2020 (7500 cases, 700 deaths). A 78% increase of hospital-onset carbapenem-resistant *Acinetobacter* spp. infections was identified between 2019 and 2020 in the Centers for Disease Control and Prevention (CDC) preliminary analysis [29]. Two Italian studies also noticed an increase in carbapenem-resistant *A. baumannii* (CRAB) colonization or infection in coronavirus disease 2019 (COVID-19) ICUs. In a study conducted in 16 Italian ICUs in the Piedmont region during the COVID-19 pandemic, 19% of patients with COVID-19 either became colonized or infected by CRAB during their ICU stay, resulting in a 67% mortality rate [30]. In another Italian study conducted in 3 medical centers, the incidence rate ratios of colonization and infection with CRAB increased by 7.5- and 5.5-fold, respectively, between January to April 2019 and January to April 2020 [27]. An MLST analysis showed that all 21 CRAB strains isolated between January and April 2020 belonged to the CC92/IC2 clonal lineage. According to the Oxford MLST scheme, ST195 (n = 15) was the most frequently identified ST, followed by ST369 (n = 6). In another US-based study evaluating 150 CRAB isolates collected from 120 patients in 4 medical centers, the authors noted that most isolates belonged to CC2 [31]. Three CC2 sublineages were identified among these isolates, with most colistin- and/or cefiderocol-resistant isolates grouped in one of the lineages. In addition, the authors also observed the emergence of ST499. The increased number of *A. baumannii* infections during the COVID-19 pandemic has also been reported in several geographic regions [32–34].

## RESISTANCE MECHANISMS

*Acinetobacter baumannii-calcoaceticus* complex isolates are naturally resistant to penicillins. As the adaptative nature of this organism is unrivalled, it is not uncommon to find isolates belonging to this species complex that are resistant to many or all available antimicrobial agents [35]. *A. baumannii-calcoaceticus* can acquire resistance to clinically used agents by horizontal gene transfer (HGT) or natural transformation [5, 36]. In addition, the acquisition of mutations or transpositions of genetic elements, such as insertion sequence *Acinetobacter baumannii* (IS*Aba*)-type elements that modulate the expression of constitutive genes encoding for enzymes, efflux systems, or outer membrane proteins, can consequently cause resistance to several antimicrobial agents.

### Exogenous Resistance Acquisition

As with other gram-negative organisms, HGT plays an important role in expanding the antimicrobial-resistance mechanisms in ABC isolates. In a study analyzing the genomes of 2 *A. baumannii-calcoaceticus* isolates, Fournier et al [36] observed an 86 kb DNA sequence that harbored a cluster of 45 resistance genes in the first isolate (a clinical isolate). For the most part, the genes observed in this DNA fragment belonged to other gram-negative genera such as *Pseudomonas*, *Salmonella*, and *Escherichia*. The authors named this genetic island AbaR1. AbaR1 contained multiple class 1 integrons carrying genes encoding β-lactamases, aminoglycoside-modifying enzymes, and efflux pumps as well as genes conferring resistance to chloramphenicol, rifampin, sulfonamides, and trimethoprim in addition to genes encoding resistance to detergents, dyes, and heavy metals. The second isolate was collected from human lice and harbored a smaller DNA region flanked by transposons in the same location as the first isolate. This structure did not contain resistance genes but did include genes derived from environmental bacterial species. Further investigations demonstrated that other complex transposons carrying resistance genes have been observed inserted into the same location [37] and that the AbaR-type resistance island is associated with IC1 while a Tn*6022*-Tn*6172* backbone structure is present in isolates from IC2.

The acquisition of antimicrobial-resistance genes in *A. baumannii* has also been associated with the transfer of plasmids. One of the early reports describing *A. baumannii* plasmids described the plasmidic profiles of *A. baumannii* clinical isolates involved in a clinical outbreak [38, 39]. The isolates analyzed carried multiple plasmids, so this feature was used as a typing method for the isolates. Regardless of this study and others that also describe *A. baumannii* plasmids, the scientific community has a limited understanding of the biology of plasmids in this species compared with Enterobacterales. *A. baumannii* plasmids are unique, as they usually carry resistance genes commonly found in other plasmids (eg, *sul2*, *strA*, and *strB*) and can vary in size from 2 to 190 kb [40–42]. A polymerase chain reaction (PCR)–based replicon-typing nomenclature that grouped these plasmids into 19 groups (GR1 to GR19) was developed [43], but this effort was limited to a small sampling of plasmids and additional groups have been described after this scheme was established.

Notably, plasmids have been involved in the mobilization of carbapenemase-encoding genes among *A. baumannii* isolates. The gene encoding OXA-58 has been detected in plasmids [44]. Several reports document the coexistence of *bla*_NDM-1_ in the same plasmid as *bla*_OXA-58_ [45–47]. Hujer et al [48] described an outbreak of OXA-237–producing *A. baumannii* among 16 patients from 5 US hospitals where this carbapenemase gene was carried by plasmids. In a recent study evaluating 43 *A. baumannii* isolates from Belgium, in all isolates carrying *bla*_OXA-72_ this gene was plasmid-borne [49]. Alternatively, *bla*_OXA-23_ was carried on the chromosome by 26 of 29 isolates that harbored this gene [49]. In a study by Cerezales et al [41], the authors evaluated the plasmids carried by 3 OXA-23–producing *A. baumannii* clinical isolates from Bolivia. The gene encoding OXA-23 was not located in the plasmids, but the isolates carried plasmids ranging in size from 67 to 184 kb. Two of the plasmids observed carried antimicrobial-resistance genes, including *tetR*, *strA*, *strB*, *aac(6′)-Ian*, and *sul2*, along with numerous insertion sequences and transposon structures [41]. D’Andrea et al [50] observed that *A. baumannii* plasmids carrying *bla*_OXA-24_ appear to have DNA modules flanked by XerC/D binding sites that allow these modules to be shuffled and recombined, facilitating the spread of resistance genes. This insight was later expanded to include multiple other *A. baumannii* plasmids [51, 52].

In contrast to the many observations of carbapenem-hydrolyzing oxacillinase genes carried in plasmids, *bla*_OXA-23_ has been primarily identified in the chromosome. The mobilization of this gene seems to be mediated by transposon structures. Nigro and Hall [53] described multiple transposons carrying the OXA-23–encoding genes, including Tn*2008*, Tn*2008B*, Tn*2009*, Tn*2007*, and Tn*2006*, that are usually located at the AbaR4 structure. In all cases, the *bla*_OXA-23_ gene was flanked upstream by IS*Aba1* or IS*Aba4*, which supplies the promoter that expresses this gene.

In addition to acquiring external resistance-encoding genes by HGT, *Acinetobacter* species can acquire single-stranded DNA from the environment and integrate these fragments into their own DNA in a process called natural transformation. Despite limited evidence of natural transformation in *A. baumannii* isolates, numerous studies evaluating the natural transformation capabilities of *A. calcoaceticus* and *A. baylyi* have been published [54–58]. In 2010, Ramirez et al [59] demonstrated that an *A. baumannii* isolate was naturally competent. In 2022, Godeux et al [60] demonstrated that carbapenem resistance could be transferred by natural transformation among *A. baumannii* isolates. These authors identified recombination events leading to the acquisition of DNA fragments ranging from 13 to 123 kb after mixing the bacterial isolates, including the transfer of AbaR1, which carried multiple resistance genes [60]. The authors concluded that natural transformation, along with HGT, are important mechanisms for acquisition of resistance mechanisms. Last, also in 2022, Dong et al [61] also demonstrated that a type IV secretion system might play a role in acquisition of antimicrobial-resistance genes by showing that the uptake of antimicrobial-resistance genes is compromised once this system is disrupted.

### β-lactamases

β-lactamase enzymes are important mechanisms of resistance to β-lactam agents in gram-negative bacilli, including *Acinetobacter* spp. In general, β-lactamases are classified into 4 molecular classes—A, B, C, and D—based on conserved amino acid motifs. Classes A, C, and D include enzymes that have an active site serine, whereas class B metalloenzymes utilize 1 or 2 zinc ions at the active site [62]. Various species of *Acinetobacter* possess intrinsic β-lactamase genes, such as members of class C (AmpC; *Acinetobacter-*derived cephalosporins [ADC]) and class D (OXA) enzymes. Class C enzymes from *A. baumannii* generally hydrolyze penicillins and cephalosporins, whereas those from class D generally hydrolyze carbapenems [62]. As described above, various studies, including a well-described report by Fournier et al [36], have shown that *Acinetobacter* spp. possess a natural ability to acquire foreign DNA, which facilitates the horizontal transfer of DNA material [63]. Consequently, numerous clinically relevant acquired β-lactamase genes have been described in *Acinetobacter* spp.

### *bla*_ADC_ 

*bla*_ADC_ genes are encoded on the chromosome of ABC and *A.* genomospecies 3. *bla*_ADC_ genes are responsible for intrinsic resistance to penicillins, cephalosporins, and first-generation β-lactam–β-lactamase inhibitor combinations [64]. Unlike other intrinsic class C genes in gram-negative bacilli counterparts, *bla*_ADC_ cannot be induced in *Acinetobacter* spp. When it occurs, *bla*_ADC_ overexpression is dependent on the presence of insertion sequences (IS) mobilized upstream [5]. Previous studies reported that IS*1133*-like elements (designated IS*Aba1*) upstream of the *bla*_ADC_ β-lactamase gene provide promoter sequences that enhance the expression of downstream genes. The IS*Aba1*–*bla*_ADC-30_ combination can increase the ceftazidime and aztreonam minimum inhibitory concentration (MIC) 4- and 64-fold, respectively, in an isogenic background [65]. Moreover, the IS*Aba1*–*bla*_ADC-30_ combination demonstrated a 16-fold increase in the sulbactam MIC in an isogenic background [66]. In addition, subtle substitutions within *bla*_ADC_ can increase the hydrolysis of aztreonam and ceftazidime and/or broaden its hydrolytic spectrum to include cefepime and the carbapenems [48].

### *bla*_OXA_ 

The *bla*_OXA_ group constitutes a very diverse cluster of enzymes with a range of narrow and extended spectra of activity; some of these enzymes also possess carbapenemase activity [67, 68]. Similar to *bla*_ADC_, *A. baumannii* encodes a gene belonging to the *bla*_OXA-51_–like cluster [68, 69]. Among cephalosporin- and/or carbapenem-resistant *A. baumannii*, *bla*_OXA-51_–like genes are often associated with IS*Aba1* located upstream, which causes overexpression of the β-lactamase gene. Previous enzymatic studies demonstrated that *bla*_OXA-51_–like genes possess weak activity toward carbapenems; therefore, a resistance phenotype should not be present solely by the overexpression of this intrinsic gene [67]. However, different variants, such as those containing the I129L substitution, have increased affinity for the carbapenems with a significant increase in hydrolytic activity.

Several groups of acquired *bla*_OXA_ genes with carbapenemase activity have been detected in ABC. The first *bla*_OXA_ gene encoding a protein with carbapenem-hydrolyzing activity was detected in a clinical *A. baumannii* recovered from a patient in Scotland in 1985 [70]. The encoding gene was initially called *bla*_ARI-1_ and later renamed *bla*_OXA-23_. *bla*_OXA-23_ appears to have originated from *Acinetobacter radioresistens*, where a variant belonging to this group was detected in the chromosome beside the ATPase gene [71, 72]. The *bla*_OXA-23_–like group has been extensively detected among *A. baumannii* isolates worldwide [73, 74] and is usually located on a plasmid as part of transposons, preceded by IS*Aba1* or IS*Aba4*. In addition, the *bla*_OXA-23_–like gene is followed by a truncated *A. radioresistens*–derived ATPase gene [72]. The *bla*_OXA-23_–like genes remain highly prevalent among resistant isolates [5, 53]. Recent studies reported that *bla*_OXA-23_–like genes were present in 39.5% and 74.5% of ABC isolates from the United States and European countries, respectively [75, 76].

Two additional acquired *bla*_OXA_ genes with carbapenemase activity that are commonly observed in *A. baumannii-calcoaceticus* are *bla*_OXA-24/40_– and *bla*_OXA-58_–like genes. OXA-24, subsequently renamed OXA-40, was first detected among isolates causing an outbreak in Spain [77]. In general, these OXA-40–like enzymes can hydrolyze penicillins, but they appear to have weak activity against cephalosporins and the carbapenems. These enzymes contribute to the decreased susceptibility to carbapenems in *A. baumannii* and the high level of resistance it achieves through the presence of multiple mechanisms [78]. Recent studies have reported that *bla*_OXA-24/40_–like genes were present in 29.0% and 20.5% of ABC from the United States and European countries, respectively [75, 76]. *bla*_OXA-58_ was first detected in an *A. baumannii* isolate from France in 2003 and few variants belong to this group. Kinetic reports described a weak activity toward the carbapenems and penicillin [67], but, similar to OXA-24, OXA-58–like enzymes will confer high-level resistance when it is combined with the additional resistance mechanisms usually present in *A. baumannii* [78].

Other groups of OXA carbapenemases have been described, such as OXA-134/235 and OXA-143. OXA-134 was detected in the chromosome of an *Acinetobacter lwoffii* and *Acinetobacter schindleri* during a study searching for progenitors of *bla*_OXA_ carbapenemases in *Acinetobacter* species other than ABC [79, 80]. OXA-235, OXA-236, and OXA-237, which share 85% identity to OXA-134, were detected in *A. baumannii* isolates from the United States and Mexico [81]. Moreover, OXA-143 was initially detected in *A. baumannii* clinical isolates from Brazil, but was observed later in Honduras, Korea, and Peru [67]. OXA-231, a D224A variant of OXA143, was initially reported in 2012 in Brazil and restricted to this region so far [82].

### Metallo-β-lactamases

Currently, metallo-β-lactamase (MBL) genes tend to be less prevalent than acquired *bla*_OXA_ carbapenemase genes in *A. baumannii.* However, many MBL genes have been detected in these species, including *bla*_IMP_, *bla*_VIM_, *bla*_SIM_, and *bla*_NDM_, and these genes cause decreased susceptibility to all β-lactam agents, except for monobactam. *bla*_IMP_ was first detected in an imipenem-resistant *Pseudomonas aeruginosa* clinical strain from Japan in 1988 [83], but several IMP variants were later described in *Acinetobacter* spp. isolates [83–93]. *bla*_IMP_ genes have been often detected in class 1 integron structures located in both the plasmid and the chromosome [94]. *bla*_VIM_ genes were first detected in *P. aeruginosa* in Italy and France in 1997 [95, 96], and since then, many studies have reported the detection of *bla*_VIM_ variants in *Acinetobacter* spp., including *bla*_VIM-1_, *bla*_VIM-2_, *bla*_VIM-3_, *bla*_VIM-4_, and *bla*_VIM-11_ [88, 93, 97–101]. Similar to *bla*_IMP_ variants, *bla*_VIM_ genes were located as a part of integron structures [96]. *bla*_SIM-1_ was first detected in *A. baumannii* isolates from Korea in 2003; this MBL type seems to be a less common carbapenemase gene in *Acinetobacter* spp. [96]. Since its first report, *bla*_SIM-1_ was detected in *Acinetobacter pittii* and *Acinetobacter nosocomialis* from Korea [102] as well as *Acinetobacter bereziniae* from Korea and *Acinetobacter baylyi* from China (GenBank entries; Kim et al 2011, unpublished data). In most, if not all, instances, *bla*_SIM-1_ was observed as part of an integron. The newest MBL type discovered, *bla*_NDM_, was detected in a carbapenem-resistant *Klebsiella pneumoniae* isolated in India in 2008 [102]. This MBL type is reported to be spread globally in various gram-negative species, with the *bla*_NDM-1_ through *bla*_NDM-5_ alleles reported in *Acinetobacter* spp. [102], as well as *bla*_NDM-7_, *bla*_NDM-14_, *bla*_NDM-40_, and *bla*_NDM-42_ (Kim et al 2011, unpublished data). Overall, the dissemination of *bla*_NDM_ in gram-negative organisms occurred almost exclusively through horizontal plasmid transfers, although *bla*_NDM_ variants were found in a variety of genetic contexts, suggesting multiple mechanisms involved in mobilization. However, in all cases, IS*Aba125* is present upstream of *bla*_NDM_ and further downstream when detected in *Acinetobacter* spp., a configuration of a composite transposon (Tn*125*). As IS*Aba125* is prevalent in *Acinetobacter* spp., it has been proposed that the IS*Aba125*–*bla*_NDM_ combination occurred initially in *Acinetobacter* spp. and later transferred to other gram-negative bacilli [103]. Among the most recent Food and Drug Administration–approved antimicrobials, only cefiderocol exhibits antimicrobial activity against CRAB and this agent has improved hydrolytic stability against β-lactamases, including carbapenemases. However, it was demonstrated that *bla*_NDM_ reduced the cefiderocol susceptibility [104].

### Other Carbapenemases and β-Lactamases in *Acinetobacter* spp.

Many other narrow- and extended-spectrum β-lactamases (NEBL and ESBL, respectively) have been detected in *Acinetobacter* spp.; however, the clinical importance of such enzymes is less clear. Numerous *bla*_CTX-M_, *bla*_SHV_, and *bla*_TEM_ sequences in *Acinetobacter* spp. were deposited in GenBank, as were sequences containing the carbapenemase gene *bla*_KPC_, mostly *bla*_KPC-2_ and *bla*_KPC-3_ [103, 105, 106]. The prevalence of such genes in *Acinetobacter* spp., especially NEBL and ESBL, is not well understood, but reports have begun to surface with the broader use of genome sequencing and in silico analysis to screen for β-lactamases. Other β-lactamases, such as *bla*_PER_, *bla*_VEB_, and *bla*_GES_, were also reported in *Acinetobacter* spp., and the prevalence of *bla*_PER_ seems to be higher than *bla*_VEB_ and *bla*_GES_. GenBank entries for *bla*_PER_ in *Acinetobacter* spp. included isolates recovered from various countries worldwide [107–111], whereas fewer entries are currently present for *bla*_VEB_ and *bla*_GES_ variants in *Acinetobacter* spp. Among these non-carbapenemase enzymes, *bla*_PER_ reduced significantly the cefiderocol susceptibility but does not appear to affect sulbactam-durlobactam [112, 113].

### Current Scenario of β-lactamases in *Acinetobacter* spp

The SENTRY Antimicrobial Surveillance Program for 2020–2021 included 788 *Acinetobacter* spp. clinical isolates collected from US hospitals and 943 collected from European countries, including Turkey and Israel. A total of 26.0% (205) and 61.8% (583) of isolates from the United States and Europe were not susceptible to imipenem and/or meropenem. These isolates were sequenced and screened in silico for β-lactamase genes. *bla*_OXA_ carbapenemase genes prevailed in this collection. All European isolates but 1 each from Israel and Turkey carried *bla*_OXA-23_–like (80.5%), *bla*_OXA-24_–like (16.6%), *bla*_OXA-213_–like (0.3%), or a combination of 2 *bla*_OXA_ carbapenamases (2.2%) (Table 1). Isolates from US hospitals also showed a similar profile, with *bla*_OXA-23_–like (52.2%) and *bla*_OXA-24_–like (29.3%) as most prevalent, but a higher number of isolates (13.2%) negative for *bla*_OXA_ carbapenemases. MBL genes were not detected in these isolates, except for 7 (1.2%) isolates carrying *bla*_NDM-1_ from Belgium (1), Germany (1), Israel (4), and Turkey (1) and 2 (1.0%) isolates from the United States (data not shown). In addition, all but 1 isolate carrying *bla*_NDM-1_ also carried *bla*_OXA-23_ (7) or *bla*_OXA-58_ (1; data not shown). A small number of isolates showed other β-lactamases, such as 8 isolates with *bla*_CTX-M-115_ (and also *bla*_OXA-24_ from Turkey), 2 isolates with *bla*_GES-1_ (and also *bla*_OXA-23_ from Turkey), 1 isolate with *bla*_GES-22_ (and also *bla*_OXA-24_ from Germany), 4 isolates with *bla*_PER-1_ (and also *bla*_OXA-23_ from Turkey), 2 isolates with *bla*_PER-7_ (and also *bla*_OXA-23_ from Turkey), and 4 isolates from the United States with *bla*_SHV-12_ and without a *bla*_OXA_ carbapenemase.

**Table 1.** Distribution of Acquired *bla*_OXA_ Carbapenemases in Carbapenem Nonsusceptible *Acinetobacter baumannii-calcoaceticus* Complex Collected as Part of the SENTRY Antimicrobial Surveillance Program (2020–2021)

OXA Genes^a^	Number of Isolates (%) by Region		Total
	Europe	United States	
OXA-23	469 (80.5)	107 (52.2)	576 (73.1)
OXA-24	97 (16.6)	60 (29.3)	157 (19.9)
OXA-23, OXA-24	4 (0.7)	5 (2.4)	9 (1.1)
OXA-23, OXA-58	9 (1.5)	0 (0)	9 (1.1)
OXA-213	2 (0.3)	2 (1.0)	4 (0.5)
OXA-134	0 (0)	3 (1.5)	3 (0.4)
OXA-58	0 (0)	1 (0.5)	1 (0.1)
Negative	2 (0.3)	27 (13.2)	29 (3.7)

Data from JMI Laboratories (data on file).

Only *bla*_OXA_ carbapenemases are represented here; additional β-lactamases could have been detected, including in isolates negative for *bla*_OXA_ carbapenemases. Additional information is provided in the text.

### Efflux Overexpression

Efflux systems, also known as efflux pumps or multidrug transporters, can confer a multidrug-resistance phenotype to the bacterial cell when overexpressed [114]. These systems are usually able to remove various classes of antimicrobial agents and other undesirable substances from the cell interior. Efflux systems can be subdivided into distinct families, including the major facilitator superfamily (MFS), the small multidrug resistance protein (SMR), the multidrug and toxic compound extrusion (MATE), and the resistance-nodulation-cell division (RND) family. RND-type efflux pumps are the most common class of multidrug transporters among gram-negative organisms [115]. Beyond the transporting protein, these systems have a membrane fusion protein (MFP) and an outer membrane protein (OMP) that allows for drug transport across both the inner and the outer membranes of gram-negative bacteria [116].

Several efflux systems have been described in ABC, including AdeABC, AdeIJK, and AdeFGH from the RND superfamily; AbeM from the MATE superfamily; and AbaF, AmvA, CraA, Tet(A), Tet(B), and Tet(X) from the MFS superfamily (Table 2). These efflux systems have different substrates and characteristics; thus, their overexpression can cause varying resistance levels. However, MIC results for certain agents might be only modestly elevated without other resistance mechanisms present [117].

**Table 2.** Efflux Systems in *Acinetobacter baumannii-calcoaceticus* Complex

Efflux Family Efflux System	Antimicrobial Class/Agent Substrate	Reference	
RND			
AdeABC	Amikacin, gentamicin, kanamycin, netilmicin, tobramycin, norfloxacin, ofloxacin, pefloxacin, sparfloxacin, chloramphenicol, cefotaxime, erythromycin, tetracycline, trimethoprim	Magnet et al [118]	
AdeFGH	Chloramphenicol, clindamycin, fluoroquinolones, trimethoprim, and decreased susceptibility to tetracycline, tigecycline, and sulfonamides	Coyne et al [127]	
AdeIJK	β-lactams, chloramphenicol, tetracycline, erythromycin, lincosamides, fluoroquinolones, fusidic acid, novobiocin, rifampin, trimethoprim	Damier-Piolle et al [124]	
MATE			
AdeM	Fluoroquinolones	Su et al [128]	
MFS			
Tet(A)	Tetracycline [overexpression of *tet*(A) might cause tigecycline resistance]	Guardabassi et al [134]	
Tet(B)	Tetracycline and minocycline	Guardabassi et al [134]	
Tet(X)	All tetracyclines, including tigecycline	He et al [136]	
AbaF	Fosfomycin	Sharma et al [129]	
AmvA	Erythromycin	Rajamohan et al [130]	
CraA	Chloramphenicol	Roca et al [131]	

Abbreviations: MATE, multidrug and toxic compound extrusion; MFS, major facilitator superfamily; RND, resistance-nodulation-cell division.

#### AdeABC

The main efflux system described in ABC is AdeABC. This efflux pump was initially described by Magnet et al [118] from an ABC isolate displaying aminoglycoside resistance. The authors noted that the AdeA-encoding gene displayed 34–39% identity, the lowest with MexX and highest with MtrC, with corresponding genes from other RND-type efflux systems. In contrast, AdeB showed 45–53% identity, the lowest with AcrD and the highest with MexD.

The AdeB component is the transporter and expels antibiotics out of the cell, while AdeA is the MFP and AdeC is the OMP [119]. The genes encoding AdeA, AdeB, and AdeC are juxtaposed and co-transcribed [120], despite the apparent independent transcription of *adeC*. The expression of AdeABC is regulated by the AdeRS 2-component system that is adjacently located and transcribed in the opposite direction of *adeABC*. AdeS is a histidine kinase sensor and AdeR is its response regulator [120]. The presence of AdeS was deemed essential for the expression of AdeABC, but the same could not be determined for AdeR, since its relationship with AdeS prevents the successful disruption of the regulator. Montana et al investigated the genetic variability of the AdeRS 2-component system in tigecycline-resistant ABC. The authors observed a higher genetic variability on *adeS* compared with *adeR*, but both genes displayed significant sequence variability and included residues deemed impactful to multidrug resistance [121]. Marchand et al [120] noted that spontaneous mutants displayed AdeS alterations in the residue 153 (Thr→Arg) located in the H box that contained the conserved histidine residue that is the site for the autophosphorylation that contributes to the increased expression of AdeABC.

The substrates of AdeB can range from hydrophilic to hydrophobic and can be either positively charged or neutral [122]. This efflux system increased the MIC values for the aminoglycosides, including amikacin, gentamicin, kanamycin, netilmicin, and tobramycin, and the quinolones, including norfloxacin, ofloxacin, pefloxacin, and sparfloxacin. Additionally, this efflux system also increased MIC values to chloramphenicol, cefotaxime, erythromycin, tetracycline, and trimethoprim when an isolate with this pump was compared with an isolate for which this pump was disrupted [118].

#### AdeIJK

The AdeIJK efflux system was the second RND efflux system to be described in ABC isolates [123]. The genes encoding this RND efflux system are organized in an operon similarly to AdeABC and are co-transcribed. In an initial report, the authors did not find a regulator that controlled the expression of AdeIJK and concluded that its expression was controlled by global regulators [124]. More recently, a TetR-type transcriptional regulator was demonstrated to control the expression of AdeIJK [125]. This gene, named *adeN*, included the disruption or alterations of its α9 helix that restored the susceptibility of its isolate to several antimicrobial agents.

As with other RND efflux systems, AdeI is the MFS, AdeJ is the pump, and AdeK is the OMP. AdeIJK-preferred substrates are amphiphilic compounds that include β-lactams, chloramphenicol, tetracycline, and erythromycin, but not azithromycin, lincosamides, fluoroquinolones, fusidic acid, novobiocin, rifampin, and trimethoprim in addition to dyes and detergents [126]. In a study by Leus et al [117], the authors constructed RND efflux-deficient isolates. They noticed that the deletion of AdeIJK caused the isolates to become hypersusceptible to almost all antimicrobial agents tested, thereby confirming the role of this efflux system in multidrug resistance. Notably, when AdeIJK and AdeABC are overexpressed simultaneously, they can confer resistance to minocycline and tigecycline [123].

#### AdeFGH

The RND efflux system AdeFGH was detected in a single-step mutant exposed to chloramphenicol or norfloxacin [127]. The strain used for this experiment was defective of AdeABC and AdeIJK, which allowed the effect of AdeFGH to be observed. These mutants had the same level of resistance to chloramphenicol, clindamycin, the fluoroquinolones, and trimethoprim, but had decreased susceptibility to the sulfonamides, tetracycline, and tigecycline.

The components of AdeFGH had less than 40% identity with AdeABC and AdeIJK, but 50% to 79% similarity with efflux systems from *Burkholderia pseudomallei* and *Burkholderia cenocepacia* [127].

The genes encoding AdeFGH are part of an operon, and the expression of this operon is controlled by an LysR-type transcriptional regulator located upstream and transcribed in the opposite direction of the genes encoding the components of the pump [127].

#### AbeM

AbeM is the only MATE efflux system conferring antimicrobial resistance reported in ABC isolates. This efflux pump increased MIC values to ciprofloxacin, gentamicin, norfloxacin, and ofloxacin. The hydrophilic fluoroquinolones, such as norfloxacin and ciprofloxacin, were better substrates for this pump [128].

#### AbaF

The literature has reported the use of fosfomycin in combination with minocycline or the polymyxins for the treatment of ABC infections that are refractory to single agents. The efflux pump AbaF and the MFS efflux system are responsible for the extrusion of fosfomycin from ABC cells and its intrinsic resistance to this antimicrobial agent [129]. AbaF is negatively regulated by AbsR25 and, in this species, AbaF has been associated with increased biofilm formation and virulence.

#### AmvA

The AmvA is an MFS efflux system in ABC isolates that has been associated with resistance to detergents, disinfectants, and dyes, in addition to tolerance to some agents [130]. This efflux system increased erythromycin MIC values 4-fold, indicating that it plays a potential role in the intrinsic resistance of ABC against this agent.

#### CraA

The CraA (chloramphenicol resistance *Acinetobacter*) MFS efflux pump described by Roca et al [131] was associated with high MIC values to chloramphenicol in ABC. CraA is structurally related to the MdfA system in *Escherichia coli* that recognizes a broad spectrum of compounds [132]. Foong et al [133] investigated a broader range of substrates, concluding that CraA was also responsible for the extrusion of thiamphenicol, florfenicol, ethidium, dequalinium, chlorhexidine, benzalkonium, mitomycin C, and the lipophilic cation TPP+.

#### Tet Family

In addition to the constitutive efflux systems, ABC isolates can acquire genes encoding efflux pumps, including Tet(A), Tet(B), and Tet(X) which cause resistance to different compounds in the tetracycline class. Tet(A) confers resistance to tetracycline while Tet(B) confers resistance to both minocycline and tetracycline. In an initial report, Guardabassi et al [134] identified that, among 35 *A. baumannii* clinical isolates, 16 carried *tet*(A) and 17 harbored *tet*(B); however, other studies documented different rates of these genes [135]. Tet(A) overexpression has been associated with tigecycline resistance in *A. baumannii* isolates that also overexpressed RND-efflux systems. Notably, Tet(X)-type encoding genes were initially observed in animal and clinical isolates in a prospective screening of Chinese *E. coli* and *A. baumannii* isolates [136]. Tet(X)-type encoding genes were deemed to cause resistance to tigecycline. The authors detected *tet*(X3) and *tet*(X4), but the prevalence of these genes was low. A later study from the same group reported *tet*(X5) in another Chinese *A. baumannii* isolate [137]. Many reports in the literature describe Tet(X) genes in livestock in China, but 1 study reported a clinical outbreak by a tigecycline-resistant, Tet(X6)-producing *A. baumannii* cluster in Taiwan [138]. Studies that attempted to find *tet*(X) genes among tigecycline-resistant isolates from other geographic regions were unsuccessful [139, 140].

### Outer Membrane Proteins

*Acinetobacter baumannii* cells appear to have a smaller number of OMPs in comparison to other gram-negative organisms. Additionally, *A. baummannii* OMPs are at least 5% smaller than the OMPs from other gram-negative organisms, making these organisms less permeable [122]. Sato and Nakae [141] measured the permeability coefficient in *A. baumannii* and observed that the permeability of cephalosporin agents is reduced 2- to 7-fold when compared with *P. aeruginosa* cells. These authors concluded that intrinsic antimicrobial-resistance levels could be attributed to this reduced permeability [141]. Decreased permeability and a low level of constitutive expression of 1 or more active efflux systems that reduces the accumulation of antimicrobial agents in the *A. baumannii* cells could account for the intrinsic resistance to several antimicrobial agents [119].

The main OMPs involved in the development of antimicrobial resistance in *A. baumannii* are CarO and OmpA. The OmpA porin is associated with decreased MICs of aztreonam, chloramphenicol, colistin, imipenem, gentamicin, nalidixic acid, and trimethoprim. The CarO porin is associated with resistance to the carbapenems [142].

OmpA is a nonspecific, abundantly expressed OMP. Iyer et al [143] demonstrated that OmpA can selectively enable the uptake of durlobactam (ETX2514), imipenem, and sulbactam, among other small molecules. These authors also demonstrated that OmpA expression increases bacterial fitness and plays an essential structural role. In a recent study, Zhong et al [144] demonstrated that the OmpA C-terminal domain can anchor β-lactamases such as OXA-23 and GES-11 in the periplasmatic space.

CarO, a 29-kDa OMP, was initially described to be a member of a novel family of β-barrel OMPs exclusively observed in members of the same phylogenetic family and class of *Acinetobacter* spp., the *Moraxellaceae* family of the class γ-Proteobacteria [142]. Resistance to carbapenems occurs due to the disruption of the CarO protein by insertion sequences [145] or from conformational changes caused by mutations in this gene [146].

### Target Site Alterations

In general, antibiotic resistance due to target site alterations appears to be more common in gram-positive organisms. For example, penicillin resistance in *Streptococcus pneumoniae* is due to mutations in penicillin-binding proteins (PBPs), β-lactam resistance in *Staphylococcus aureus* is due to PBP2a mutations, and oxazolidinone resistance is due to mutations in 23S ribosomal RNA (rRNA) [147–149]. However, target site alterations can also confer antibiotic resistance in gram-negative bacilli. For example, there are point mutations in GyrA and ParC that result in fluoroquinolone resistance. Also, a 4-amino-acid insertion within PBP3 in *E. coli* causes resistance to many β-lactam agents, including new agents such as ceftazidime-avibactam and aztreonam-avibactam, when combined with other resistance mechanisms [150].

Previous publications have reported imipenem resistance in *A. baumannii* to be associated with PBP alterations [151, 152] or overexpression of PBP genes [152]. Other studies reported the reduced expression of a 73-kDa PBP, or PBP1b [153]. As described above, resistance to fluoroquinolones in *A. baumannii* was reported to be caused by mutations within the quinolone-resistance determinant region of GyrA, GyrB, and ParC [154]. Aminoglycosides are agents commonly used in combination therapies for treating *A. baumannii* infections. In general, aminoglycoside resistance occurs in *A. baumannii* due to the presence of aminoglycoside-modifying enzymes [155]; however, genes coding for 16S rRNA methyltransferases generate a target site alteration/methylation and high-level resistance to aminoglycosides, including plazomicin, in *A. baumannii* [156–158].

Last, rifampin and polymyxin compounds are often used in combination treatment for *A. baumannii* infections, especially recently with the increasing number and dissemination of resistant clinical isolates causing life-threatening diseases [159]. It was reported that resistance to rifampin was associated with point mutations at the target site, RpoB [160, 161]. Similarly, polymyxin resistance in *A. baumannii* has been mostly associated with alterations in composition and structure of the lipopolysaccharide (LPS) layer of the cell envelope, the target site of the polymyxins. In many other gram-negative species, the reduction in the negative charge of the LPS is mediated by the addition of 4-amino-4-deoxy-l-arabinose (AraN) to the lipid A, which leads to poor binding of the polymyxin molecules. Differently, in *A. baumannii*, the addition of phosphoethanolamine (PetN) regulated by the PmrAB two-component systems (TCS) seems to be the most common colistin resistance mechanism [162]. Mutations in the cognate regulator PmrA can autoregulate the TCS operon. In addition, mutations in *pmrB* are also seen in *A. baumannii*. The loss or inactivation of the LPS has also been described in *A. baumannii*. These changes are mediated by mutations within the *lpxA*, *lpxC*, and *lpxD* genes [161, 163]. Last, Trebosc et al [164] reported that the insertion of the IS*AbaI* element upstream of the PmrC homologue EptA can lead to colistin resistance in clinical isolates.

Beyond colistin resistance, hetero-resistance is often reported in *A. baumannii*. In a review by Cai et al [165], the authors concluded that hetero-resistance rates surpass the resistance rates and can vary from 18% to 100% depending on the study and methodology used for the detection of hetero-resistance, while resistance rates varied from 0% to 40%. The mechanisms for hetero-resistance are unclear; however, there have been reports of reversible resistance due to acquisition of compensatory mutations or reversion of the mutated genotype [162].

Cefiderocol resistance in *A. baumannii* remains complex and not very well understood. TonB-dependent transporters provide uptake of siderophore–iron complexes throughout the bacterial outer membrane. Previous studies showed that cefiderocol resistance in *A. baumannii* can be caused by alterations within the TonB operon (*tonB-exbB-exbD*). The insertion of IS*Aba1* at the C-terminus of *tonB* was found to cause reduced expression of *exbB* and *exbD* genes in resistant mutants [166]. Similarly, frameshift mutations in components of the inner membrane protein complex in *exbD3* or *tonB3* genes led to significant increases in the MICs of siderophore–iron complexes [167]. Other studies reported that reduced or absent expression of the TonB receptors *pirA* and/or *piuA* was associated with cefiderocol resistance [168–170]. In addition, resistance to cefiderocol was also linked to alteration within PBP3 [168].

## CONCLUDING REMARKS

*Acinetobacter baumannii-calcoaceticus* complex is a clinically significant pathogen, especially in the nosocomial setting. This organism represents a therapeutic challenge due to the limited treatment options available for infections caused by it. The global spread of *A. baumannii* IC complexes that exhibit multiple mechanisms of resistance is responsible for the high multidrug-resistance rates observed worldwide. Among the mechanisms conferring elevated MIC results to agents that are otherwise active against *A. baumannii* isolates, carbapenemases of the OXA family are the leading cause of carbapenem and β-lactam resistance in this species. Additionally, target alteration and efflux-mediated resistance are often reported in *A. baumannii* as a cause of resistance to important agents used to treat infections due to these organisms, including polymyxins, cefiderocol, and other β-lactams.

A significant increase in the number of CRAB infections was noticed during the COVID-19 pandemic in many ICUs, mainly attributable to the increasing demand for healthcare and the lack of adherence to the prevention and infection-control polices [171]. The treatment of serious CRAB infections still represents a clinical challenge, and efficacious therapies, whether traditional or nontraditional therapies, are urgently needed [172].

## Notes

***Acknowledgments.*** The authors thank Amy Chen, PhD, for the editorial assistance.

***Financial support*.** This work was sponsored by Entasis Therapeutics, a wholly owned subsidiary of Innoviva, Inc. The authors did not receive any fees for authorship.

***Supplement sponsorship*.** This article appears as part of the supplement “Sulbactam-durlobactam, a Targeted β-lactam/β-lactamase Inhibitor, for MDR *Acinetobacter*,” sponsored by Entasis Therapeutics Inc., a wholly owned subsidiary of Innoviva, Inc.
